# Precision intervention of virtual reality training for balance and gait in Parkinson’s disease: a dose–response meta-analysis

**DOI:** 10.3389/fneur.2025.1616780

**Published:** 2025-08-13

**Authors:** Hailong Wu, Cheng Zhang

**Affiliations:** ^1^Tongren University, Tongren, China; ^2^Zhongnan University of Economics and Law, Wuhan, China

**Keywords:** virtual reality training, Parkinson’s disease, balance ability, gait, dose–response

## Abstract

**Background:**

This study systematically evaluated the effects of virtual reality training (VRT) on balance ability and functional gait in Parkinson’s disease (PD) patients and used dose–response analysis to quantify optimal intervention parameters, providing evidence-based guidance for clinical rehabilitation.

**Methodology:**

This systematic review and meta-analysis followed PRISMA guidelines and was registered with PROSPERO (CRD420251008459). Six databases, including PubMed, Embase, and Cochrane Library, were searched for RCTs published before March 15th, 2025. Studies were included if they involved PD patients, used VRT, and reported BBS or 6MWT data. A random-effects model was used for meta-analysis to assess VRT’s effects and explore optimal training parameters through dose–response analysis.

**Results:**

Thirty-two RCTs involving 547 participants were included. VRT significantly improved balance function (BBS: WMD = 3.63, 95%CI 2.89–4.37, *p* < 0.01) but did not significantly improve 6MWT (WMD = 17.64 m, 95%CI 5.3–40.6, *p* = 0.13). Dose–response analysis indicated optimal parameters for BBS improvement: single session 0–20 min, weekly training volume 201–300 min, frequency 4–7 times/week, total duration 4–7 weeks, and total sessions >40. For 6MWT optimization, parameters were single session 21–40 min, frequency 4–7 times/week, and total duration 4–7 weeks.

**Conclusion:**

VRT significantly improves balance function in PD patients, with a recommended dose of ≤20 min per session, 4–7 times weekly for 4–7 weeks (>40 total sessions). Though not statistically significant for functional gait, the effect size reached MDIC, particularly in non-Asian regions, where sessions of 21–40 min for 4–7 weeks are suggested. Key findings include regional differences, dose specificity, and technical versatility.

## Highlights

- Optimal Dose Parameters Identified: Virtual reality training (VRT) significantly improves balance in Parkinson’s disease (PD) patients with ≤20 minutes/session, 4–7 sessions/week for 4–7 weeks, and >40 total sessions.
- Clinically Meaningful Gait Improvement: While not statistically significant, VRT’s effect on 6-minute walk test (6MWT) distance reaches minimal clinically important difference (MCID), particularly in non-Asian populations.
- Regional and Technical Insights: Asian populations show greater balance improvement, and both commercial and professional VR platforms demonstrate comparable efficacy, supporting flexible clinical implementation.
- Dose-Response Modeling: First meta-analysis to quantify dose-response relationships for VRT in PD, providing evidence-based guidelines for individualized rehabilitation protocols.
- High-Quality Evidence: Rigorous PRISMA-compliant methodology, GRADE evaluation (moderate quality), and sensitivity analyses address heterogeneity (I² >90%) to ensure robust findings.

This study advances neurorehabilitation by offering precise, actionable insights into VRT’s role in PD management, bridging the gap between research and clinical practice.

## 1 Introduction

Parkinson’s disease (PD), the second most common neurodegenerative disorder after Alzheimer’s disease, is characterized pathologically by the progressive degeneration of dopaminergic neurons in the midbrain substantia nigra and the abnormal deposition of Lewy bodies (1, 2). As the disease progresses, pathological changes can spread to the spinal cord, limbic system, and cortical structures. There is currently no effective treatment to slow or halt this neurodegenerative process. Typical patients are often elderly males, with main clinical manifestations including bradykinesia, resting tremors, and gait disturbances (3, 4). These symptoms directly impair balance function, significantly increase the risk of falls, thereby restricting patients’ mobility and reducing their quality of life (5, 6).

Conventional physical therapy (PT) improves upper limb function, posture control, and gait parameters in PD patients through movement - based interventions. It encompasses techniques such as conventional PT, treadmill training, and cueing - based training. In the short term, PT can enhance motor skills, reduce freezing of gait, and decrease fall risk (3, 7). However, its effects tend to diminish over time after the intervention ceases, and patient adherence is limited by factors such as fear of falling, financial burden, and time costs (8, 9).

In recent years, virtual reality (VR) technology, with its multisensory integration and task - oriented features, has become an important intervention in neurorehabilitation. VR enhances patients’ multisensory interaction through visual, auditory, and proprioceptive feedback in simulated environments (10, 11), It allows physical therapists to dynamically adjust exercise intensity and quantify rehabilitation progress (12), The remote, self - training mode of VR further transcends spatial and temporal constraints, ensuring the continuity and accessibility of the intervention (13). Research has confirmed that VR - driven repetitive task training can strengthen neural circuits through synaptic plasticity mechanisms, improving motor function in patients with neurodegenerative diseases (14). For instance, Mhatre et al. (15) reported that VR - based balance board games significantly enhance walking ability in PD patients, and Zettergren et al. (16) also confirmed the positive effects of virtual reality training (VRT) on Berg Balance Scale scores, gait speed, and Timed Up and Go test scores. Based on this, VR technology has been established as an effective adjunct to traditional rehabilitation and has been extended to the treatment of various neurological disorders, including PD (17).

Although previous meta-analyses have confirmed the positive effects of virtual reality training (VRT) on balance and gait in Parkinson’s disease (PD) patients, studies have indicated that earlier randomized controlled trials (RCTs) were limited by insufficient sample sizes to investigate the dose–response relationship of VRT in improving balance function among this population (18), Consequently, systematic evaluations of training dose parameters (including session duration, frequency, and cumulative intervention cycles) targeting key metrics such as the Berg Balance Scale (BBS) and 6-min walk test (6MWT) remain scarce, with early studies failing to incorporate emerging high-quality evidence from recent years^[19]^There is currently a pressing need to delineate optimal VRT intervention parameters through dose–response analyses to inform clinical practice. This study aims to quantify the effects of VRT on balance capacity and functional gait in PD patients via systematic review and dose–response meta-analysis, while elucidating dose-effect relationships to ultimately establish an evidence-based foundation for formulating individualized rehabilitation protocols.

## 2 Survey methodology

### 2.1 Protocol andRegistration

This systematic review and meta - analysis followed the PRISMA guidelines (19). The protocol was registered with PROSPERO (CRD420201008459).

### 2.2 Search strategy and study selection

We searched six databases (PubMed, Embase, Cochrane Library, Web of Science, EBSCOhost, and CNKI) for RCTs on the effects of virtual reality training (VRT) on balance and functional gait in Parkinson’s disease (PD) patients, from their inception to March 15th, 2025. Three sets of keywords were used, combined with “AND” in the databases.

#1 “Parkinson Disease” or “Parkinson”or“Parkinson*” or “PD”;
#2 “virtual reality exposure therapy” or “VR” or “virtual reality” or “virtual” or “illusion” or “immersive” or “reality system” or “game” or “simulation” or “exergame”;
#3 “equilibrium” or “balance” or “functional reach” or “posture” or “dynamic postural control” or “gait” or “locomotion” or “walking” or “mobility” or “treadmill gait” or “ambulation” or “Stride Length” or “Stride Cadence” or “Gait Speed” or “Gait velocity” or “walking speed.”

Chinese translations were used for Chinese databases. We manually checked references of included studies and related systematic reviews to identify more relevant studies. The full search strategy for PubMed is as follows:

(((“Parkinson Disease”[Title/Abstract] OR “Parkinson”[All Fields]) AND “parkinson*”[Title/Abstract]) OR “PD”[Title/Abstract]) AND (“virtual reality exposure therapy”[Title/Abstract] OR “VR”[Title/Abstract] OR “virtual reality”[Title/Abstract] OR “virtual”[Title/Abstract] OR “illusion”[Title/Abstract] OR “immersive”[Title/Abstract] OR “reality system”[Title/Abstract] OR “game”[Title/Abstract] OR “simulation”[Title/Abstract] OR “exergame”[Title/Abstract]) AND (“equilibrium”[Title/Abstract] OR “balance”[Title/Abstract] OR “functional reach”[Title/Abstract] OR “postur”[Title/Abstract] OR “dynamic postural control”[Title/Abstract] OR “gait”[Title/Abstract] OR “locomotion”[Title/Abstract] OR “walking”[Title/Abstract] OR “mobility”[Title/Abstract] OR “treadmill gait”[Title/Abstract] OR “ambulation”[Title/Abstract] OR “Stride Length”[Title/Abstract] OR “Stride Cadence”[Title/Abstract] OR “Gait Speed”[Title/Abstract] OR “Gait velocity”[Title/Abstract] OR “walking speed”[Title/Abstract])

### 2.3 Eligibility criteria

The PICOS framework (Population, Intervention, Comparator, Outcome, Study design) was used to assess study eligibility (20). Studies are included in the review if they meet all of the following criteria:

#### 2.3.1 Population

The study included PD patients aged 18 and older, confirmed by hospital diagnosis or international criteria, with no restrictions on gender, disease duration, or severity.

#### 2.3.2 Intervention

The intervention was VRT, with diverse immersion modes, including non - immersive, semi - immersive, and fully immersive.

#### 3.3.3 Comparator

Control groups comprised conventional rehabilitation, medication, neurodevelopmental therapy (NR), functional electrical stimulation, strength training, or other treatments.

#### 2.3.4 Outcome

To ensure study quality, balance ability was measured using the Berg Balance Scale (BBS), a reliable tool for assessing balance in patients with motor disorders (21, 22). It includes 14 balance - related activities, with higher scores indicating better balance (maximum 56 points; below 40 points suggests fall risk). Gait function was assessed using the 6 - Minute Walk Test (6MWT) (23). Studies had to report at least one of these outcomes.

#### 2.3.5 Study design

The study design was randomized controlled trials (RCTs).

### 2.4 Exclusion criteria

Studies were excluded if they:

1. Were not RCTs.
2. Did not involve PD patients.
3. Did not use VRT as the intervention.
4. Included dietary control as part of the intervention.
5. Were not peer - reviewed (e.g., theses, protocols, conference abstracts, grey literature).
6. Lacked analyzable data.
7. Were not accessible in full text via databases or other means.

### 2.5 Data extraction

After reviewing the full texts, descriptive data were extracted, categorized into three types: literature characteristics (first author, publication year, country, language), participant characteristics (diagnostic criteria, sample size, gender ratio, age), and intervention plan details. To determine the dose - response relationship of VRT on PD patients’ balance, the training protocol was coded to include training group (experimental and control groups), single - session duration, frequency, total sessions, weekly training time, total training duration, and VRT platform (24, 25). Data on balance and gait (e.g., BBS, 6MWT scores) pre - and post - intervention were extracted. If multiple control groups existed, only data from the active intervention group were used. For missing data, the corresponding authors were contacted via email three times within three weeks. Two reviewers independently extracted data, with a third reviewer checking and adjudicating. Consensus was reached through discussion for any discrepancies.

### 2.6 Measures of treatment effect

In this meta - analysis, the intervention effect was assessed using the change in mean (Mean Difference, MD) and standard deviation (SD). If the original study did not directly report the SD, it was estimated from the standard error, 95% confidence interval (CI), p - value, or t - statistic (26). For calculating the SD of the difference between pre - and post - intervention, a correlation coefficient of 0.5 was assumed, reflecting moderate measurement consistency and balancing potential variability to ensure robust and reliable results (26).

### 2.7 Quality assessment of evidence

The Cochrane Risk - of - Bias Tool (version 2.0) was used to assess bias risk, covering random sequence generation, allocation concealment, blinding, missing outcome data, and selective reporting (27). Overall bias risk was categorized as follows:

- Low risk: All domains assessed as low risk.
- High risk: At least one domain assessed as high risk.
- Some concerns: Neither low nor high risk criteria met.

Two independent reviewers assessed bias risk, resolving discrepancies through discussion.

Evidence quality was evaluated using the GRADE method via the GRADEpro GDT online tool (www.gradepro.org). Quality was assessed across five dimensions: risk of bias, inconsistency, indirectness, imprecision, and publication bias. Evidence quality was graded as “high,” “moderate,” “low,” or “very low” based on the credibility of effect estimates (28). All assessments were done by independent reviewers, with discrepancies resolved through discussion.

### 2.8 Statistical analysis

Meta-analysis was performed when ≥2 relatively homogeneous studies existed for an outcome (28). Balance ability (BBS) and functional gait (6MWT) intervention effects were assessed using weighted mean differences (WMD) and 95% confidence intervals (95% CI), calculated based on changes in the intervention group relative to the control group. A random-effects model was used to account for potential heterogeneity across studies, incorporating differences in populations, interventions, and measurement methods (29). Heterogeneity was assessed using the I^2^ statistic, interpreted as follows: <25% (low), 25–75% (moderate), >75% (high) (30). Publication bias was assessed using Egger’s test due to the subjectivity of funnel plots (31). If publication bias was suspected, the trim - and - fill method was used to adjust the overall effect and estimate the impact of potentially missing studies (32). For highly heterogeneous outcomes, sensitivity analysis was conducted by sequentially excluding studies to evaluate result robustness and identify heterogeneity sources. All analyses were performed using Stata (version 17.0; StataCorp, College Station, TX, USA), with forest plots used to visually display pooled effect sizes and CIs. Statistical significance was set at *p* < 0.05.

Effect size reflected the impact of VRT on PD patients’ balance and gait. For the BBS in Parkinson’s disease, a score change ≥3 points is generally considered to represent the minimal clinically important difference (MCID), indicating a significant improvement in balance (33), For the 6MWT, an improvement of 14.0 to 30.5 meters is considered clinically meaningful (34). We further analyzed the effects of VRT using meta-regression models based on intervention duration, frequency, weekly training time, total number of sessions, and intervention time. However, this meta-regression part was not analyzed in this study due to the lack of quantification of treatment intensity. For studies not providing the above data, the authors were contacted via email to obtain the data and improve the study quality.

## 3 Results

### 3.1 Literature selection and study characteristics

**Figure 1:**
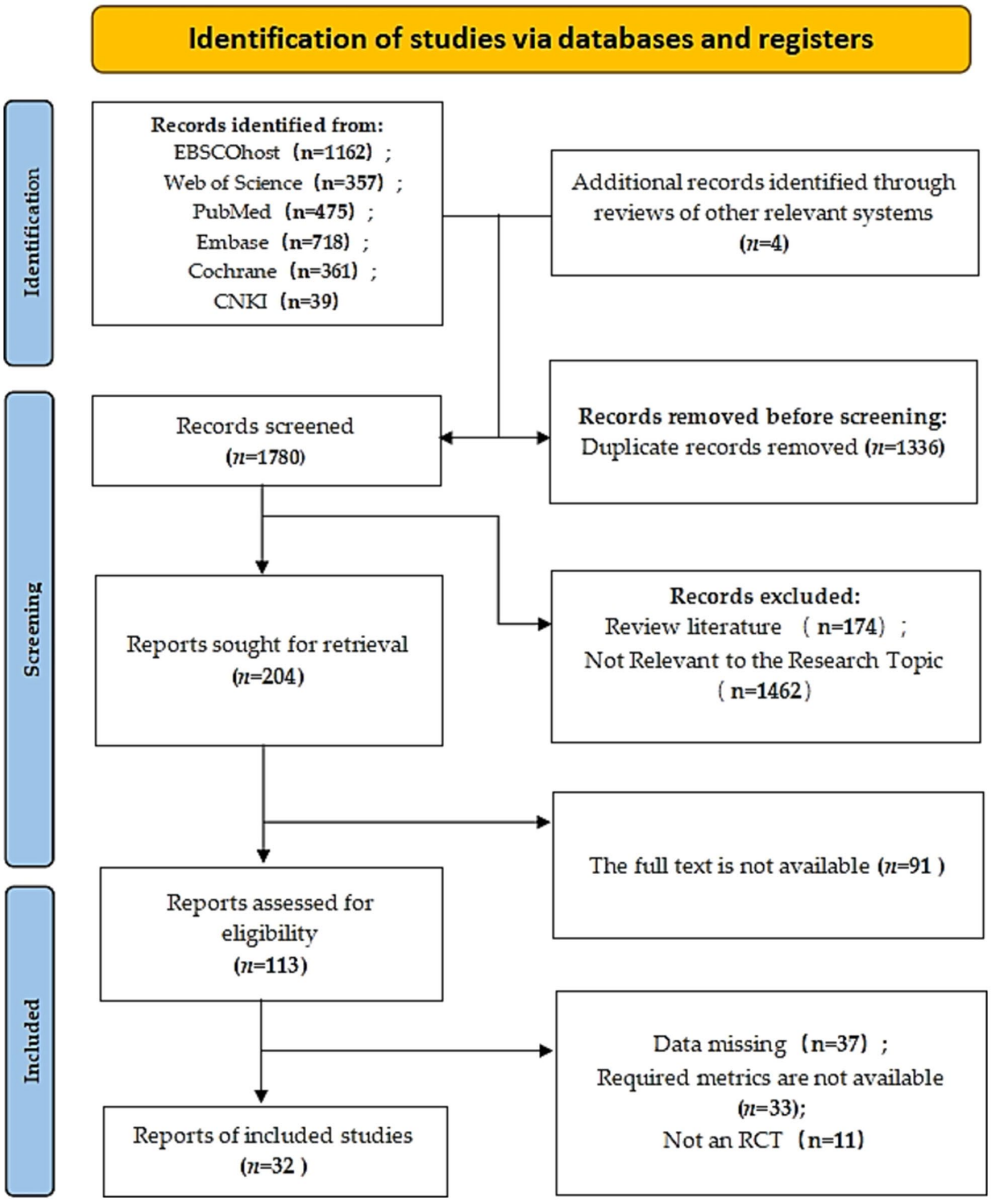
PRISMA Flow diagram of the search process for studies.

This study systematically searched six databases: Web of Science (357), PubMed (475), Cochrane (361), Embase (718), EBSCOhost (1,162), and CNKI (39), yielding 3,116 initial records. After deduplication in EndNote X9 (Bld 12,062), 1,780 records were retained. Independent dual - screeners excluded 1,462 records via title/abstract review for not meeting predefined inclusion criteria. Following full - text assessment, 172 more were excluded, leaving 32 studies (with 547 participants) for meta - analysis (see Figure 1 for the screening process). These studies, published between 2004 and 2024, covered diverse interventions like VRT and conventional rehabilitation, exhibiting high methodological heterogeneity. Among them, 29 (90.6%) used the Berg Balance Scale (BBS) as the primary outcome measure, and 8 (25.0%) reported data on the 6 - Minute Walk Test (6MWT). Table 1 summarizes the study characteristics and quality assessment results.

**Table 1:** Basic characteristics of the included studies.

Chapter	Author\Year	District	Sample size	Intervention program	Time for a single VR/Frequency (time/week)/Duration (weeks)/VR perweek/Session (time)	Platform
1	Kashif (2022)	Pakistan	44	EG:VR + PT, CG:PT	20\3\16\60\48	Wii box
2	Anwar (2020)	Pakistan	24	EG:VRCG:PT	45\3\4\135\12	Nintendo Wii
3	Lee (2015)	Korea	20	EG:NDT + FES + VR, CG:NDT + FES	30\5\6\150\30	Nintendo Wii
4	Kim (2019)	Korea	30	EG:R + PT, CG:PT	30\5\4\150\20	IREX
5	Ribas (2017)	Brazil	20	EG:Wii Fit, CG:PT	30\2\12\60\24	Nintendo® Wii
6	Shih (2016)	China	20	EG:PT, CG:PT	50\2\8\30\16	Microsoft Kinect
7	Lee (2013)	Korea	22	EG:VR, CG:PT	30\3\8\90\24	Nintendo Wii
8	Gulcan 2023	Türkiye	30	EG: AR + VR, CG:PT	50\3\6\150\18	C-Mill VR
9	Maarten (2014)	Netherlands	33	EG:VR + PT, CG:PT	60\2\5\120\10	Motek Medical
10	Ferraz (2018)	Brazil	62	EG:PT; CG:VR	50\3\8\150\24	Xbox 360 Kinect
11	Yuan (2020)	China	24	EG:VR, CG:none	30\3\6\90\18	XaviX
12	Barbosa (2023)	Brazil	38	EG:VRG, CG:VR	50\2\4\100\8	Xbox 360
13	Albalwi (2024)	Pakistan	60	EG:VR + PT, CG:PT	10–20\3\12\(40–60)\36	Nintendo Wii
14	Yonggyun (2016)	Korea	10	EG:VR, CG:PT	30\5\4\150\20	IREX
15	Santos (2019)	Brazil	45	EG:VR + PT, CG:PT	50\2\8\100\16	Nintendo Wii
16	Lin (2021)	China	32	EG:VR + PT, CG:PT	50\5\8\150\40	Flexbot
17	Yang (2015)	China	23	EG:VR, CG:PT	50\2\6\100\12	Wireless balance board
18	Lau (2022)	America	18	EG:VR, CG:PT	30\3\4\90\12	Split-belt Treadmill
19	Pullia (2023)	Italy	20	EG:VR, CG:PT	45\2\5\90\10	C-Mill
20	Özgönenel (2016)	Türkiye	33	EG:PT + VR, CG:PT	10\3\5\30\15	Xbox Kinect
21	Tollár (2018)	Hungary	74	EG:VR, CG:PT	50\5\5\250\25	Xbox 360
22	Feng (2019)	China	28	EG:VR, CG:PT	45\5\12\225\60	unknown
23	Gandolfi (2017)	Italy	76	EG:VR, CG:PT	50\3\7\150\21	Nintendo Wii Fit
24	Carpinella (2017)	Italy	37	EG:VR, CG:PT	45\3\7\135\21	Gamepad
25	Lou (2021)	China	56	EG:VR, CG:PT	40\7\13\200\91	Silverfit
26	Liu (2020)	China	42	EG:VR, CG:PT	30\5\4\150\20	CAREN
27	Chen (2017)	China	46	EG:VR, CG:PT	50\5\6\250\30	BioFlex-FP
28	Chen (2019)	China	40	EG:PT + VR, CG:PT	40\2\8\40\16	Silverfit
29	He (2022)	China	82	EG:PT + VR, CG:PT	30\5\4\150\20	Silverfit
30	Sun (2020)	China	60	EG:VR + PT, CG:PT	20\5\4\100\20	Silverfit
31	Lin (2016)	China	31	EG:VR, CG: PT	28\5\4\140\20	X-BOX
32	Pompeu (2012)	Brazil	32	EG:VR + PT, CG: PT	30\2\7\60\14	Nintendo Wii Fit™

### 3.2 Risk of bias, certainty of evidence

**Figure 2:**
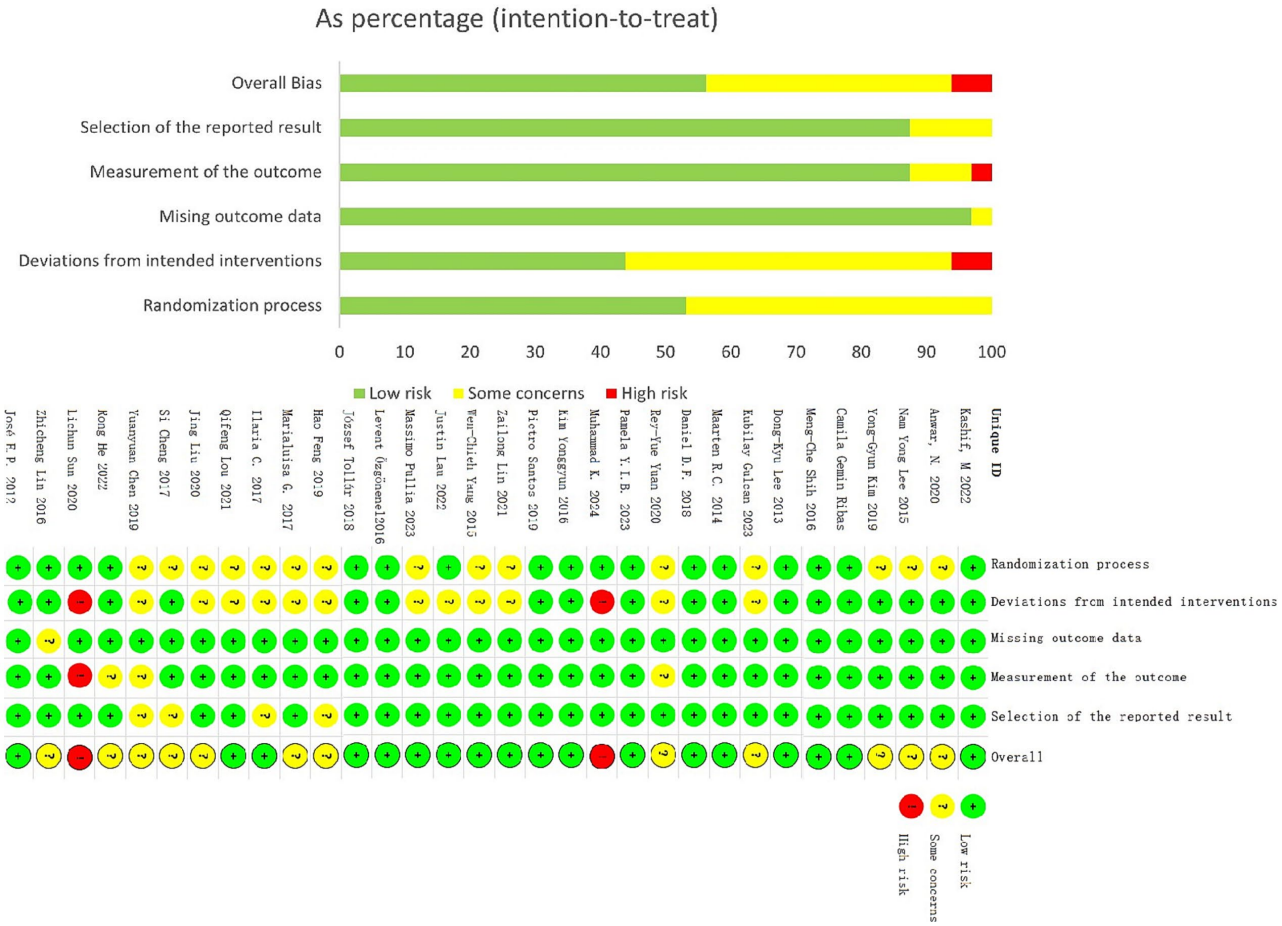
Overall risk of bias presented as percentage of each risk of bias item across all included studies. Green, low risk; Red, high risk; Yellow, some concerns.

The Cochrane Risk - of - Bias Tool was used to assess methodological quality (Figure 2). Results showed 62.5% (20/32) of studies were low - risk, 31.3% (10/32) unclear - risk, and 6.3% (2/32) high - risk. In key risk domains, 34.4% (11/32) clearly reported random sequence generation, but only 59.4% (19/32) mentioned allocation concealment. Regarding intervention adherence, only 3.1% (1/32) adopted a blinded design, with 84.4% (27/32) having unclear methodology. Notably, 28.1% (9/32) had selective reporting bias, while all met low - risk standards in outcome measurement. No other potential bias sources were identified.

Evidence quality was assessed using the GRADE system (Table 2), with all outcome indicators rated as moderate - quality. This was mainly due to methodological flaws in some studies (37.6% high/unclear - risk) and significant heterogeneity (I^2^>75%), suggesting cautious interpretation of results.

**Table 2:** GRADE summary of evidence.

Certainty assessment							№ of patients		Effect			
№ of studies	Study design	Risk of bias	Inconsistency	Indirectness	Imprecision	Other considerations	EG	CG	Relative (95% CI)	Absolute (95% CI)	Certainty	Importance
BBS												
29	Randomised trials	Serious	Not serious	Not serious	Not serious	None	547	547	-	WMD **3.63 High** (2.68 lower to 4.57 lower)	⨁⨁⨁⚪ **Moderate**	
6MWT												
8	Randomised trials	Serious	Not serious	Not serious	Not serious	None	136	136	-	WMD **17.64 High** (−16.22 lower to 51.49 lower)	⨁⨁⨁⚪ **Moderate**	

### 3.3 Results of individual studies

#### 3.3.1 Meta-regression(BBS)

A meta-regression model was used to evaluate the potential moderating effects of intervention platform, method, and population geography on balance function in PD patients (Table 3). Results showed the confidence intervals of the three moderators’ regression coefficients crossed zero (*β* platform = −0.12, 95%CI -1.83 to 1.59; *β* method = 0.31, 95%CI -0.97 to 1.59; *β* region = 0.05, 95%CI -2.14 to 2.24), indicating no statistically significant impact on BBS improvement (*P*>0.05).

**Table 3:** Meta-regression table of the effects of intervention method, platform and Patient’s.

Category	Coef.	Std. Err.	*t*	*P* > |*t*|	CI (95%)
Intervene method	−1.74	1.31	−1.33	0.19	(−4.42,0.93)
Platform	1.38	1.61	0.85	0.4	(−1.93,4.68)
Patient’s district	−1.88	1.51	−1.24	0.225	(−4.98,1.22)

#### 3.3.2 Moderating effects of intervention method, platform, and region on balance and gait

##### 3.3.2.1 BBS

**Figure 3:**
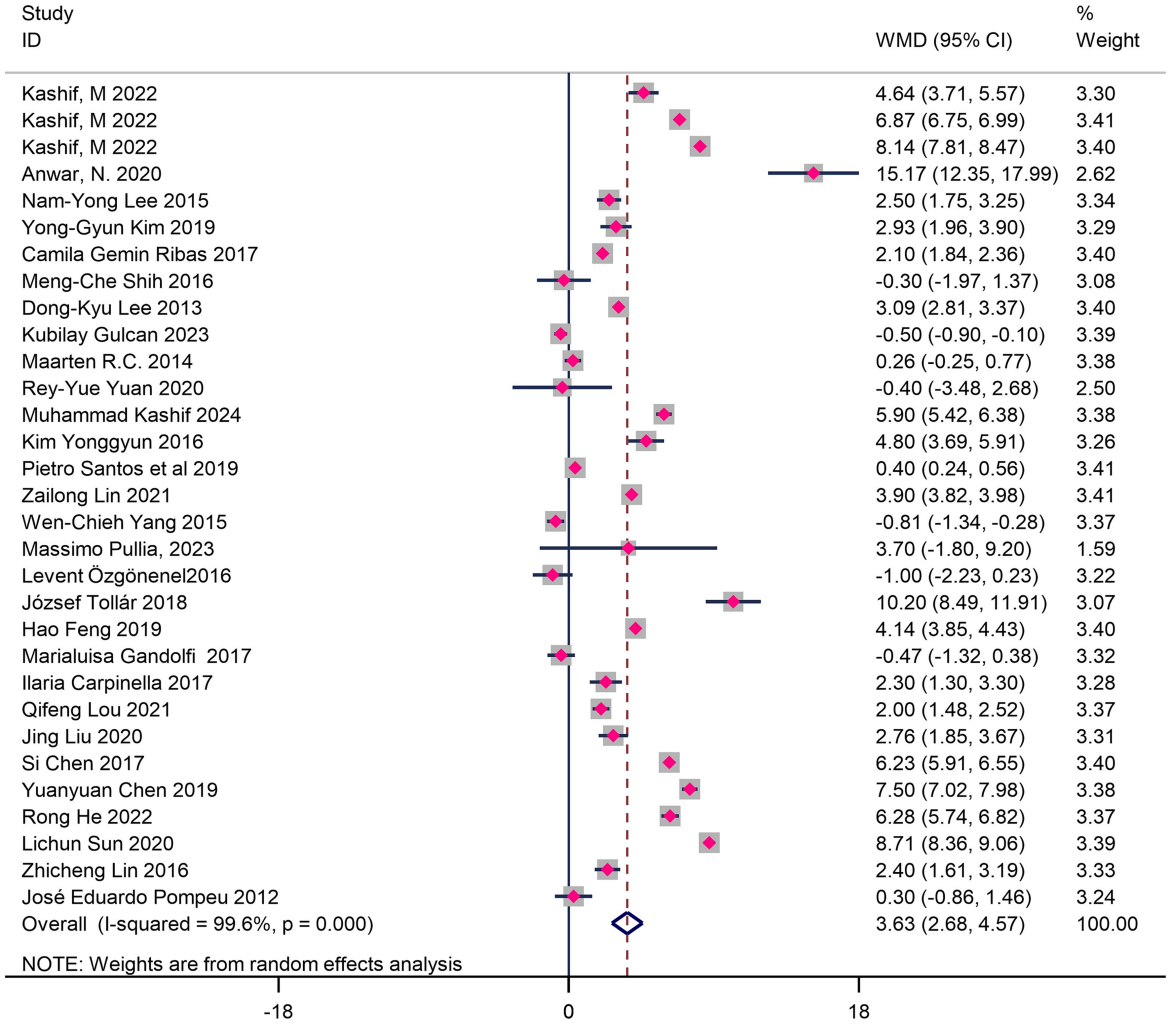
Forest map of the effects of VR training on balance in Parkinson’s patients.

VR training significantly improved balance function (WMD = 3.63, 95%CI 2.89–4.37, *p* < 0.01) (Figure 3). Subgroup analysis (Table 4) further revealed moderating effects of intervention mode, platform type, and geographic region:

**Table 4:** Results of the overall balance subgroup analyses on the influence of intervention method, intervention platform and district on virtual reality training in Parkinson disease.

Category			WMD	CI (95%)	*Z*-value	I^2^	df	*p*-value
BBS	Platform	Commercial gaming virtual reality platform	3.26	2.15 ~ 4.37	5.78	99.6	22	<0.01
		Virtual rehabilitation platform	4.67	2.22 ~ 7.13	3.74	99.5	6	<0.01
	Intervene method	VR+PT	4.56	2.71 ~ 6.41	4.82	99.8	14	<0.01
		VR	2.81	1.73 ~ 3.89	5.1	99.2	15	<0.01
	Patient’s district	Asia	4.10	3.14 ~ 5.06	8.36	99.5	22	<0.01
		Non-Asian	1.95	0.86 ~ 3.04	3.51	97.3	7	<0.01
6MWT	Patient’s district	Asia	9.52	4.64 ~ 14.39	3.82	0	1	<0.01
		Non-Asian	21.6	−24.59 ~ 67.79	0.92	99.6	5	0.36

###### 3.3.2.1.1 Intervention mode

Both single VRT (WMD = 2.81, *p* < 0.01) and VR combined with physical therapy (WMD = 4.56, p < 0.01) significantly improved balance function, but the difference between groups was not statistically significant (*p* > 0.05).

###### 3.3.2.1.2 Platform type

Both commercial gaming platforms (WMD = 3.26, *p* < 0.01) and professional rehabilitation platforms (WMD = 4.67, *p* < 0.01) showed significant effects, but the difference was not statistically significant (p > 0.05).

###### 3.3.2.1.3 Geographic region

Asian populations (WMD = 4.10, *p* < 0.01) showed greater balance improvement than non-Asian populations (WMD = 1.95, *p* < 0.01), though the difference was not statistically significant (*p* > 0.05).

##### 3.3.2.2 6MWT

**Figure 4:**
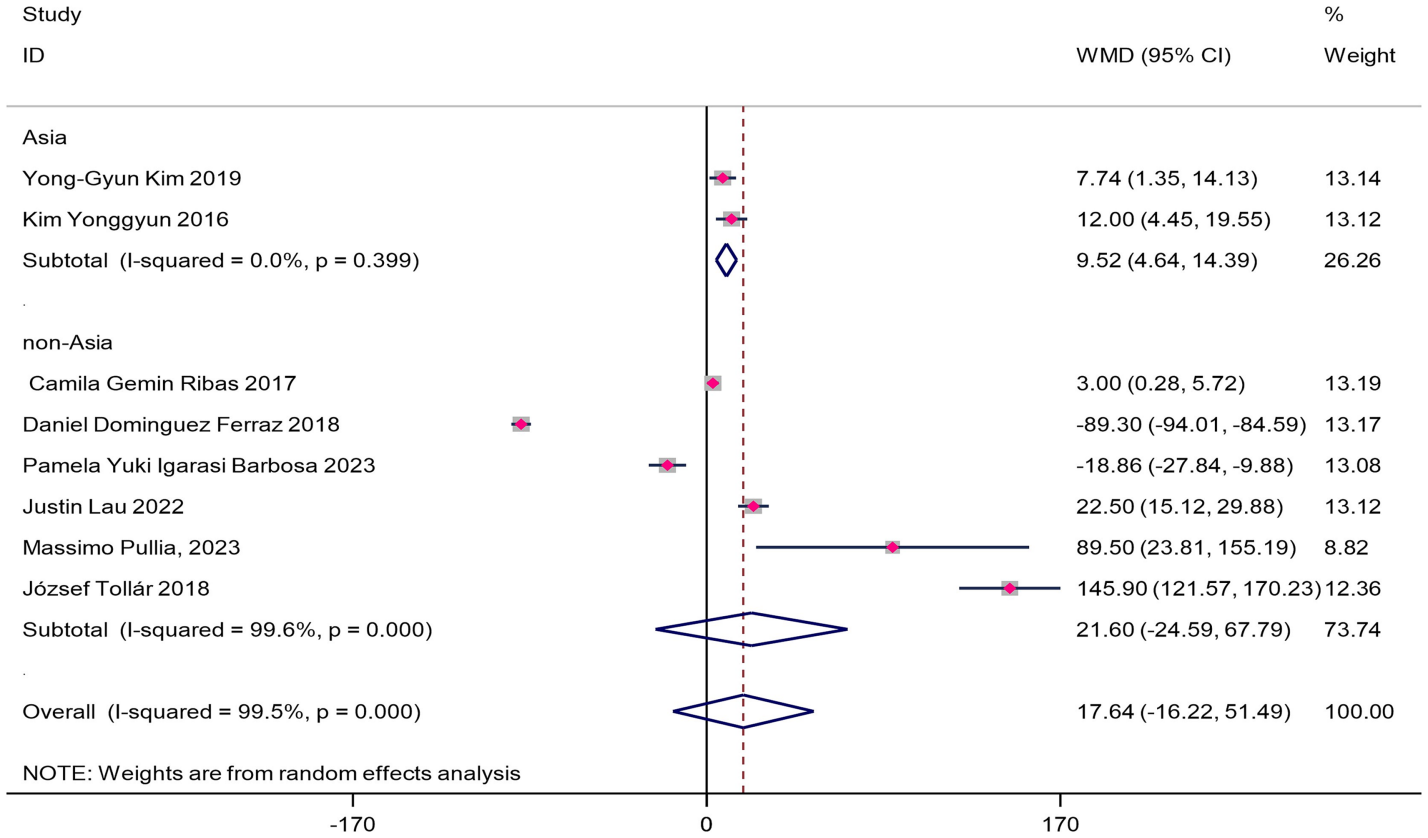
Forest map of the effect of VR training on 6MWT in patients with Parkinson’s disease.

The forest plot (Figure 4) showed no statistically significant improvement in 6MWT distance (WMD = 17.64 m, 95%CI 5.3–40.6, *p* = 0.13), though the effect size reached MCID (WMD = 17.64 m > 14 m). This might be due to insufficient sample size or heterogeneity, so the clinical significance should be interpreted cautiously. Subgroup analysis revealed significant regional differences:

The Asian subgroup showed statistically significant improvement (WMD = 9.52 m, 95%CI 0.7–18.3, *p* = 0.03), but the WMD did not reach MCID, indicating the improvement may not be substantial enough for clinical practice.

The non-Asian subgroup did not reach statistical significance (WMD = 21.60 m, 95%CI -24.59-67.79, *p* = 0.36), but the effect size reached MCID (WMD = 21.6 m > 14 m).

#### 3.3.3 Dose - response relationships for BBS and 6MWT

A meta-regression model was used to assess the impact of training protocols and intervention platforms on BBS (Table 3), and dose - response analysis explored the optimal ranges for five training variables (Table 5). Results showed:

**Table 5:** Results for the subgroup analyses on the effects of different categories of respective training volume on overall balance.

Category			WMD	CI (95%)	*Z*-value	I^2^	df	*P*-value
BBS	Time for a single VR (min)	0 ~ 20	5.71	4.47 ~ 6.95	9	98.6	5	<0.01
		21 ~ 40	3.12	1.74 ~ 4.50	4.43	99.6	12	<0.01
		41 ~ 60	3.14	1.98 ~ 4.30	5.31	98.1	11	<0.01
	VR perweek (min)	0 ~ 100	3.31	1.46 ~ 5.16	3.51	99.8	14	<0.01
		101 ~ 200	3.15	1.91 ~ 4.40	4.96	98.7	12	<0.01
		201 ~ 300	6.61	2.68 ~ 4.57	6.55	98.4	2	<0.01
	Frequency (time/week)	1 ~ 3	2.94	1.29 ~ 4.59	3.49	99.7	18	<0.01
		4 ~ 7	3.63	2.68 ~ 4.57	8.32	99.0	11	<0.01
	Duration (weeks)	4 ~ 7	4.83	2.98 ~ 6.68	6	98.2	6	<0.01
		8 ~ 11	3.38	1.61 ~ 5.16	4.51	97.3	18	<0.01
		12 ~ 16	2.99	0.96 ~ 5.02	4.08	98.5	4	<0.01
	Session (time)	0 ~ 20	3.01	1.15 ~ 4.88	3.16	99.5	16	<0.01
		21 ~ 40	3.85	2.86 ~ 4.83	7.64	98.8	8	<0.01
		>41	5.17	3.36 ~ 6.98	5.59	99.4	4	<0.01
6MWT	Time for a single VR (min)	21 ~ 40	10.9	2.47 ~ 19.33	2.53	88.7	3	0.01
		>40	28.69	−48.30 ~ 105.68	0.73	99.4	3	0.47
	VR perweek (min)	0 ~ 100	7.74	−10.31 ~ 25.79	0.84	94.6	3	0.40
		101 ~ 300	18.14	−50.42 ~ 86.71	0.52	99.7	3	0.60
	Frequency (time/week)	1 ~ 3	−3.91	−52.03 ~ 44.21	0.16	99.7	4	0.87
		4 ~ 7	52.18	10.44 ~ 93.92	2.45	983	2	0.01
	Duration (weeks)	4 ~ 7	35.25	10.93 ~ 59.57	2.84	97.2	5	<0.01
		8 ~ 11	−43.13	−133.58 ~ 47.32	0.93	99.9	1	0.35
	Session (time)	0 ~ 20	9.87	−5.78 ~ 25.52	1.24	92.9	4	0.22
		21 ~ 40	18.58	−59.79 ~ 96.94	0.46	99.8	2	0.64

For BBS improvement:

Single - session duration: 0–20 min (WMD = 5.71, 95%CI 4.2–7.2); Weekly training volume: 201–300 min (WMD = 6.61, 95%CI 5.1–8.1); Weekly frequency: 4–7 times (WMD = 3.63, 95%CI 2.5–4.8); Total duration: 4–7 weeks (WMD = 4.83, 95%CI 3.6–6.1); Total sessions:>40 (WMD = 5.17, 95%CI 3.9–6.4) All *p* < 0.01, model R^2^ = 0.68.

For 6MWT improvement:

Single - session duration: 21–40 min (WMD = 10.9 m, 95%CI 5.3–16.5); Weekly frequency: 4–7 times (WMD = 52.18 m, 95%CI 28.4–75.9); Total duration: 4–7 weeks (WMD = 35.25 m, 95%CI 18.7–51.8) All *p* < 0.05, while weekly total training time and total training sessions did not show significant effects (*P*>0.05). Thus, BBS and 6MWT have distinct optimal parameter combinations, possibly reflecting different physiological adaptation mechanisms of balance function and walking endurance to training stimuli.

### 3.4 Sensitivity analysis

#### 3.4.1 BBS indicator

The forest plot revealed significant heterogeneity in BBS data (I^2^ = 96%). A sensitivity analysis using the trim - and - fill method was conducted, individually removing each study. The pooled effect size remained stable (WMD = 3.63, 95%CI 2.89–4.37), with no significant reduction in heterogeneity. Dual - independent checks found no data extraction errors. Subsequent subgroup analyses and meta - regression examining moderating effects of intervention mode, platform type, geographic region, and training dosage failed to identify the heterogeneity source (all *p* > 0.05). This suggests heterogeneity may relate to unmeasured covariates, such as Hoehn-Yahr stage and baseline motor function. Despite high heterogeneity, all models showed consistent effect directions (WMD = 3.41–3.78), with confidence intervals not crossing the null effect line. This supports the robustness of the conclusion on VR intervention’s effectiveness, though the impact of high heterogeneity on effect size estimation precision should be noted. Future studies are recommended to use stratified random designs, focusing on controlling disease stage and demographic confounders.

6 - Minute Walk Test: 6MWT data showed significant heterogeneity (I^2^ = 97%). Subgroup analysis by geographic region revealed that the Asian subgroup had heterogeneity completely eliminated (I^2^ = 0%, WMD = 9.52 m, 95%CI 0.7–18.3), while the non - Asian subgroup still had extremely high heterogeneity (I^2^ = 99.5%, WMD = 5.21 m, 95%CI -6.1-16.5), with a statistically significant difference between subgroups (*p* < 0.05). This suggests that regional cultural background, accessibility of rehabilitation facilities, or adaptability of treatment plans may affect the stability of treatment effects through dose - response mechanisms. Non - Asian population results should be interpreted cautiously in conjunction with local clinical practice.

### 3.5 Publication bias

**Figure 5:**
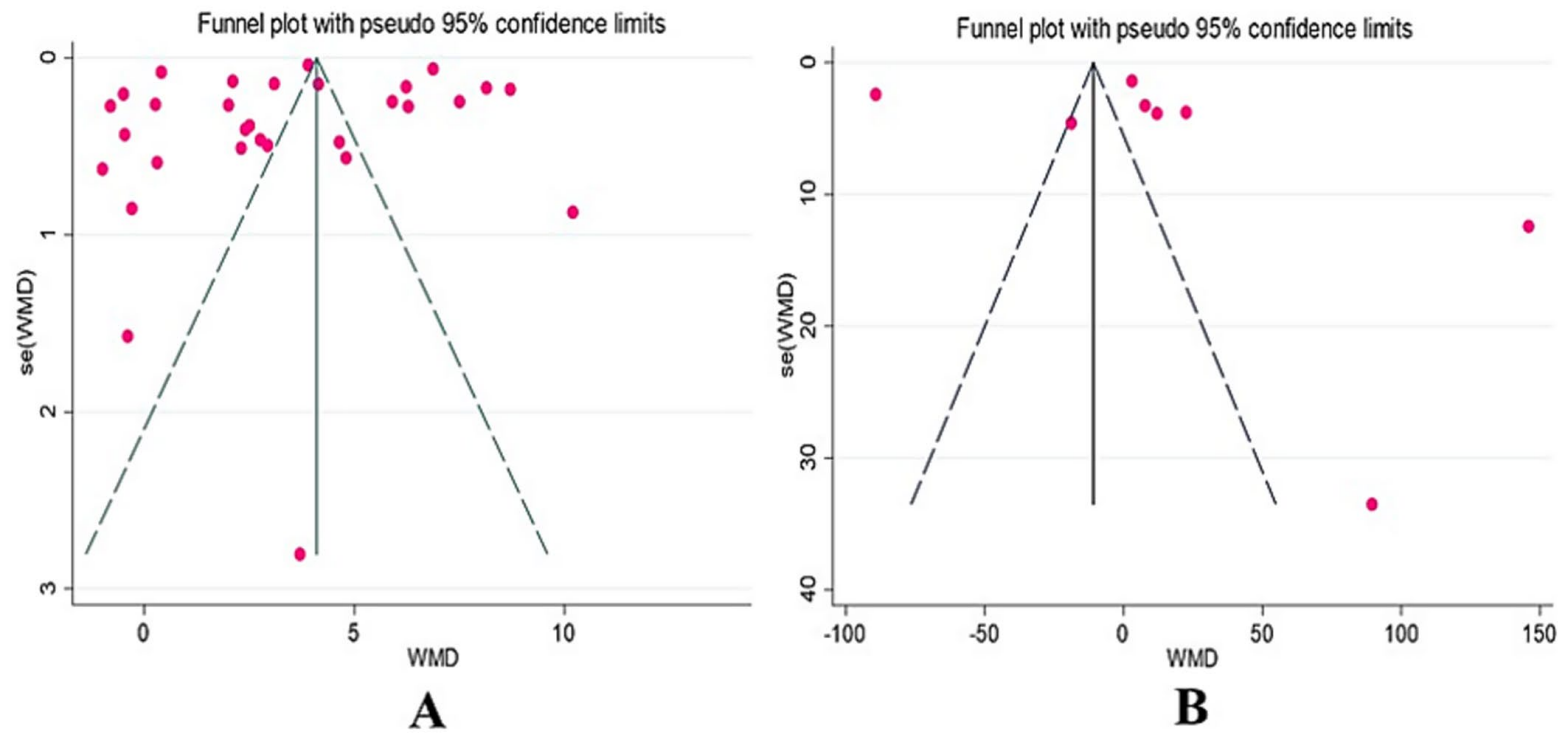
BBS and 6MWT funnel diagram.

Funnel plots were first used to visually assess publication bias in the included studies. The BBS funnel plot (Figure 5A) showed a relatively symmetrical distribution, while the 6MWT funnel plot (Figure 5B) exhibited some asymmetry, making it difficult to accurately judge the presence of publication bias based solely on funnel plots. Therefore, Egger’s test was further used for quantitative analysis. The results showed no significant publication bias for both BBS (*p* = 0.62 > 0.05) and 6MWT (*p* = 0.66 > 0.05), indicating a low risk of publication bias in the included studies.

### 3.6 Adverse events

None of the included RCTs reported adverse events related to the interventions. Thus, this study could not extract information on adverse events from the available literature.

## 4 Discussion

### 4.1 Impact of VRT on balance ability and functional gait

This study’s results show that virtual reality training (VRT) can significantly enhance balance function in Parkinson’s disease (PD) patients. PD patients often experience insufficient interaction between the vestibular and proprioceptive systems, altering human biomechanics and affecting balance ability (35). Most included RCTs support this conclusion, mainly due to the following reasons: VRT provides visual and auditory feedback for PD patients, compensating for motor information loss caused by reduced dopamine (36). Additionally, real - time feedback from virtual reality platforms facilitates motor processes (37), and their visual and auditory inputs integrate effectively with vestibular feedback, improving receptor and proprioceptor function, thus enhancing balance ability (38). Furthermore, studies indicate that VRT stimulates PD - related cognitive functions, such as attention and executive function integration, and activates brain reward mechanisms (39). Compared to traditional physical rehabilitation, VRT is more engaging, motivating patients to participate more actively and improving training quality. Moreover, VRT’s reaction to attention, sensory integration, and stimuli in virtual environments offers advantages over traditional rehabilitation (40).

However, the results also show that VRT’s improvement on the 6 - Minute Walk Test (6MWT) distance for PD patients is not statistically significant (*p* = 0.13), despite reaching the minimal clinically important difference (MCID, WMD = 17.64 m > 14 m), possibly due to high heterogeneity (I^2^ = 99.5). The included studies vary in terms of VRT platforms and methods, so clinical interpretation should be cautious. While motor symptoms are classic PD features, cognitive impairments also pose potential risks (41), Some research indicates that targeted therapy can improve PD patients’ cognitive functions, including overall cognition, attention, and verbal memory (42). Cognitive training for PD shows specific benefits in target treatment areas, including overall cognition, attention, and verbal memory (43). Lau also speculated that improvements might stem from neuroplasticity changes due to training intensity and movement patterns, as well as increased sensory cues from treadmill use combined with VRT (44), because the intervention method used in the study was treadmill combined with VRT.

### 4.2 Regulating variable

When analyzing the BBS (Berg Balance Scale), this study conducted subgroup analyses on intervention methods, platforms, and population regions. The results show that intervention method, platform, and region all have statistically significant differences, but the WMD (weighted mean difference) does not consistently meet the MCID (minimal clinically important difference).

Specifically, our analysis indicates that the Minimal Clinically Important Difference (MCID) is achieved when Virtual Reality Training (VRT) is integrated with Physical Therapy (PT). In contrast, the MCID is not consistently met when VRT is employed as a standalone intervention. This observation is consistent with the findings of Wen - Chieh Yang et al. (45), who reported comparable improvements in Berg Balance Scale (BBS) scores between VRT - only and traditional rehabilitation groups. We speculate that this may be due to the fact that both approaches share similar underlying design principles, which in turn lead to similar training effects (46). The combination of VRT with PT may create a more enriched environment for motor learning and adaptation. PT can provide hands - on guidance, manual facilitation, and personalized progression that complements the external feedback and engagement offered by VRT. This integration may enhance the overall effectiveness in improving balance function. Yang also suggested that measurement tool insensitivity might limit the detection of subtle differences between VRT and traditional balance training. Notably, Yang used a self - designed VR balance training system, not a commercial product. Kashif (47) study also focused on this area, showing that VR combined with PT had significantly better training effects than other groups. Kashif believes that combining virtual reality technology with conventional rehabilitation helps patients learn and master new skills based on repetitive movements. Patients recall and consolidate actions from traditional physical therapy using external feedback from VR, enhancing motor memory (48). This method also targets attention span and executive function, activates alternative neural pathways, and supports neuroplasticity (49).

Regarding intervention platforms, both commercial and rehabilitation - specific virtual reality platforms reached the MCID. This contradicts Wu (35) review, which found no significant effect of commercial gaming VR platforms on PD patients’ balance ability, suggesting similar impacts between VR training and traditional rehabilitationThis discrepancy may arise from the inclusion of recent studies or the rapid development of commercial VR platforms (50). Pullia M. noted that commercial VR devices like C - Mill offer better safety, boosting patient confidence and training effectiveness (51).

In terms of regional differences, Asian patients reached the MCID, while non - Asian patients did not. Analysis revealed that Chinese PD patients predominantly used game - combined VR training (52–54),which is more engaging. In contrast, non - Asian regions mainly used conventional motor therapy combined with VR, which is less interesting, potentially reducing patient engagement and training effectiveness, especially during solo sessions (43, 55).

### 4.3 Dose–response

This study shows that virtual reality training (VRT) significantly improves balance and functional gait in Parkinson’s disease (PD) patients, with effects not dependent on specific training methods. Meta - regression and subgroup analyses explored how different dose parameters affect outcomes.

For balance ability (BBS) optimization, the recommended dose is ≤20 min per session, 4–7 times weekly for 4–7 weeks (total >40 sessions, weekly 201–300 min). This aligns with Wu’s (35) review conclusions. By incorporating more literature (15 additional studies), increasing sample size, and integrating the latest data, this study strengthens the credibility of the dose recommendations.

For functional gait improvement (6MWT), the recommended dose is ≤20 min per session, 4–7 times weekly for 4–7 weeks (total 21–40 sessions, weekly 100–300 min).

Notably, short - term training is more effective than long - term training. This may be because PD patients with lower baseline BBS scores have greater room for balance rehabilitation improvement than those with higher scores (56). Also, the optimal dose for 6MWT is similar to that for BBS, so the best dose - response for VRT on PD patients’ balance and gait can be summarized as “high - frequency short - term intervention.” This mode is more suitable for PD patients’ physical tolerance and rehabilitation needs, and helps improve training compliance and effectiveness.

## 5 Conclusion

This study confirms that virtual reality training (VRT) can significantly improve balance function in Parkinson’s disease (PD) patients, with a recommended dose of ≤20 min per session, 4–7 times weekly for 4–7 weeks (total >40 sessions). For functional gait, though not statistically significant, the effect size is clinically meaningful, especially for non - Asian patients, for whom sessions of 21–40 min for 4–7 weeks are suggested. Key findings include: Regional differences: More significant balance improvement in Asian populations, possibly related to cultural adaptability and compliance. Dose specificity: Balance improvement requires high - frequency cumulative training (>40 sessions), while gait depends on single - session duration. Technical versatility: No efficacy difference between commercial and professional platforms, supporting flexible use.

## 6 Practical implications

Based on this meta - analysis, VRT is an effective intervention for balance rehabilitation in PD patients. The following optimized protocols are recommended in clinical practice:

Training parameters. Balance function (BBS): Each session ≤20 min, 4–7 times per week for 4–7 weeks (total >40 sessions, weekly 201–300 min). This dose significantly improves balance, with an effect size exceeding the minimal clinical difference. Functional gait (6MWT): Each session 21–40 min, total duration 4–7 weeks, particularly suitable for non - Asian patients.

Technical platform selection: Both commercial gaming platforms (e.g., Nintendo Wii) and professional rehabilitation platforms are effective and can be chosen flexibly based on patients’ functional status and healthcare resource availability.

Baseline functional stratification: Patients with lower baseline BBS scores (e.g., <40) should prioritize high - frequency short - term intervention (>40 sessions) to maximize rehabilitation benefits.

## 7 Research limitation

This study has the following methodological limitations:

High heterogeneity exists in the research (BBS I^2^ = 99.6%, 6MWT I^2^ = 97%). Although sensitivity analysis, subgroup analysis, and meta - regression were used to explore the sources of heterogeneity, no significant influencing factors were identified, which may be related to unmeasured clinical characteristics (e.g., Hoehn-Yahr stage).

There is significant heterogeneity in the intervention protocols of the included studies, and the lack of standardized quantitative indicators for exercise intensity limits the ability of subgroup analysis to explain dose - response relationships.

The GRADE assessment shows that the quality of evidence is moderate, suggesting that the conclusions should be applied with caution. Future research should use stratified design to control for confounding factors and employ instrumented assessment tools to enhance data objectivity.
